# Unraveling the genetic complexity underlying sorghum response to water availability

**DOI:** 10.1371/journal.pone.0215515

**Published:** 2019-04-18

**Authors:** Nguyen Phuong, Gloria Afolayan, Hartmut Stützel, Ralf Uptmoor, Mohamed El-Soda

**Affiliations:** 1 Institute of Biological Production Systems, Leibniz Universität Hannover, Hannover, Germany; 2 Department of Agronomy, University of Rostock, Rostock, Germany; 3 Department of Genetics, Faculty of Agriculture, Cairo University, Giza, Egypt; College of Agricultural Sciences, UNITED STATES

## Abstract

Understanding the adaptation mechanisms of sorghum to drought and the underlying genetic architecture may help to improve its production in a wide range of environments. By crossing a high yielding parent (HYP) and a drought tolerant parent (DTP), we obtained 140 recombinant inbred lines (RILs), which were genotyped with 120 DArT and SSR markers covering 14 linkage groups (LGs). A subset of 100 RILs was evaluated three times in control and drought treatments to genetically dissect their response to water availability. Plants with early heading date (HD) in the drought treatment maintained yield (YLD) level by reducing seed number SN and increasing hundred seed weight (HSW). In contrast, early HD in the control treatment increased SN, HSW and YLD. In total, 133 significant QTL associated with the measured traits were detected in ten hotspot regions. Antagonistic, pleiotropic effects of a QTL cluster mapped on LG-6 may explain the observed trade-offs between SN and HSW: Alleles from DTP reduced SN and the alleles from HYP increased HSW under drought stress, but not in the control treatment. Our results illustrate the importance of considering genetic and environmental factors in QTL mapping to better understand plant responses to drought and to improve breeding programs.

## Introduction

*Sorghum bicolor* L. Moench. is native to arid and semi-arid tropical environments and a drought-tolerant cereal. In general, sorghum growing seasons in Sub-Saharan Africa are characterized by initial rainfalls with subsequent periods of drought. Sorghum plants with high vigor and fast growth rates during early developmental stages may be advantageous in regions affected by drought early in the season [1]. A plant’s response to drought can be categorized into three adaptive strategies, i.e., drought escape (e.g by early flowering), drought tolerance (e.g. by improving water-use efficiency), and drought avoidance (e.g. by increasing water uptake and reducing water loss, [2–4]. Evaluating the natural variation of these responses by testing large numbers of genotypes in several environments improves the understanding of genotype by environment interactions (G×E) ([5], which in turn allows to select breeding lines with improved yield stability and helps to identify superior alleles across different environments [6]. Dissecting the genetic components underlying G×E can be achieved via mapping quantitative trait loci (QTL) and their effects in different environments, i.e., by estimating QTL by environment interaction (Q×E) effects [7–9].

The common approach to explore QxE is to use multi-environment analysis [7, 8]. However, to develop indirect selection strategies for yield via its genetically correlated components, the multi-trait multi-environment (MTME) approach [10] is superior over independent multi-environment or multi-trait approaches [11]. The power of MTME is basically due to its ability to map QTL with different effects, such as QTL with synergistic pleiotropic effects, where one allele affects two or more traits in the same direction. If the allele is antagonistically affecting two different traits, one allele improves one trait while the other allele enhances other traits. Another possibility is the conditional neutrality of a QTL, i.e. the QTL has an effect on a trait in one environment but has no effect on the same trait in other environments. Additionally, the same allele can have unequal effects on the same trait in two environments, i.e. the effect is strong in one environment but weak in other environments [9, 11]. Considering such effects in breeding programs is crucial since selecting for QTL, which are mapped in single environments, might lead to undesired responses in other environments [9].

In the present study, we focused on investigating the relation between vegetative growth of shoots and roots and yield components as well as yield. To genetically dissect these relationships, we used 100 recombinant inbred lines (RILs) genotyped with 120 DArT and SSR markers.

We used the raw data from three experiments in the present manuscript, although a transformed form of parts of the data was used earlier in a manuscript, which was submitted, published and later retracted from the Journal of Agricultural Sciences [12]. The retracted paper was published without permissions of all coauthors. Retraction was mainly done due to the mentioned fact and due to several minor errors and mistakes throughout the manuscript: Even though the data included yield components and yields, the title suggested an analysis of only vegetative traits. The discussion of the manuscript submitted earlier did not include comparisons with earlier results, which are covered in the present paper. The retracted paper did not account for interactions between genotypes and water availability. Consequently, the QTL mapping approach did not distinguish between QTL with main, conditionally neutral and antagonistic pleiotropic effects. In the present study, we overcame those limitations by using the more powerful MTME–QTL-mapping-approach.

## Materials and methods

### Developing and genotyping the RIL population

The RIL population was developed at the Grain Crops Institute (GCI), Potchefstroom, South Africa from a cross between a high yielding parent (HYP) with superior grain quality under normal conditions and strongly reduced yields under drought stress and a breeding line with intermediate yielding abilities, which was described as drought tolerant (DTP). From the F_1_, 140 RILs were advanced to F_4_ by selfing.

DNA was extracted from leaf tips of F_4_ seedlings using the cetyl-trimethylammonium bromide (CTAB) method. Genotyping was carried out at Diversity Arrays Technology Pty. Ltd. (DArT), Yarralumla, Australia using 184 polymorphic DArT markers. In addition, nine informative expressed sequence tag (EST) derived simple sequence repeat (SSR) markers [13] were used. Polymerase chain reaction (PCR) was carried out using a T-Gradient PCR machine (Biometra, Göttingen, Germany). The PCR protocol had a denaturation temperature of 94°C, an annealing temperature of 52°C, and a polymerization temperature of 72°C. The first 25 cycles with 30 s for each step were followed by eight cycles with an annealing time extended to 45 s and a polymerization time of 60 s. We used DY-682 labeled M13 primers in the PCR reactions (Eurofins MWG, Ebersberg, Germany). Amplification products were separated by polyacrylamide gel electrophoresis using an LI 4200 sequencer (Licor Inc. Lincoln, USA). The genetic map was constructed with the 193 markers using JoinMap 4 (Kyazma B.V., Wageningen, The Netherlands, www.kyazma.nl) and the multipoint maximum likelihood mapping function [14]. Low informative markers such as monomorphic markers, markers with a high number of missing scores and those with more than 75% allele skewedness towards either A or B alleles were removed.

### Experimental setup and plant phenotyping

Three experiments were carried out under controlled greenhouse conditions using a subset of 100 RILs, which were grown in a control and a drought treatment with two replications arranged in a completely randomized block design. The first two experiments were conducted in order to estimate pre-flowering drought stress effects on plant growth during the vegetative phase. The third experiment was carried out in order to estimate drought stress effects on yield and yield components. Plants grown in the control treatment were watered every second day, whereas plants grown under drought stress were watered until most plants were in the fourth-leaf stage. The greenhouse conditions were kept at average day/night temperatures of 25.8/15.9°C and mean day/night relative humidity was 37.4/65.2%. The lengths of the stress cycles were 18, 21 or 43 days, respectively, in the three experiments.

In the first experiment, two seeds were sown in each of the 12.5 x 50 cm polyvinyl chloride pots filled with 9.4 kg dry sandy soil and supplemented with 1100 ml nutrient solution, which corresponds to 80% of the maximum soil water holding capacity (WHC). After emergence, plants were thinned to a single plant per pot and fertilized with 0.15% Scotts Universal Orange (Scotts Marysville, Ohio, USA) solution (N:P:K 16:6:26) twice a week. Evaporation was minimized by covering the soil with 200 g of gravel. The second experiment was basically a replication of the first experiment, while both focused on growth and development of vegetative plant parts before flowering. The third experiment was to analyze effects of drought on grain yield and yield components.

After harvesting the first and the second experiments, leaf area (LA) was measured using a LI-3100 area meter (Licor Inc., Lincoln, NE, USA). Harvested plants were stored in plastic bags until LA measurement to minimize errors due to transpiration losses and senescence. Roots were washed carefully, placed in a water bath and scanned with a flatbed scanner. Total root lengths (TRL) were measured using WinRhizo (Regent Instruments Inc., Quebec, Canada). Dry weights of leaves, stems (SDW), and roots were measured after drying plant parts at 105°C until weight constancy. Above ground dry matter (AGDM) was calculated as the sum of LDW and SDW. Specific leaf area (SLA) was calculated as ratio between LA and LDW. For the third experiment, heading date (HD) was determined, seed number (SN) per plant was counted, hundredseed weight (HSW) was measured and (YLD) was calculated.

### Statistical analysis

Statistical analysis was performed using SPSS 2. We used the following mixed linear model with fixed environment and random genotype effects:

$$y_{ijk}=\mu +\alpha_{i}+\beta_{j}+\gamma_{ij}+\varepsilon_{i_{jk}}$$

where *μ* is the overall mean, *α*_*i*_ is the effect of the *i*th environment, *ß*_*j*_ is the effect of the *j*th genotype, *γ*_*ij*_ is the interaction effect of the *j*th genotype with the *i*th environment and *ε*_*ijk*_ is a random error.”

Broad-sense heritability for each trait was estimated as the ratio between the genetic variance *Vg*, i.e. the variance among all lines, and the total phenotypic variance *Vt*, i.e. *Vt = Vg + Ve*, where *Ve* is the error variance, i.e. the variance among replications.

### QTL mapping using the multi-trait multi-environment approach

Means of the 18 measured traits (S1 Table) were used to map QTL with Genstat 16 (VSN International, Hemel Hempstead, UK) using the multi-trait multi-environment analysis (MTME) approach [10, 11]. The whole genome was scanned by simple interval mapping (SIM) with a distance of 30 cM to separate selected QTL. To estimate the allelic effect and the explained phenotypic variance of each QTL per trait and treatment, backward selection on the significant cofactors was used.

## Results

### Linkage map construction and population phenotyping

**Fig 1:**
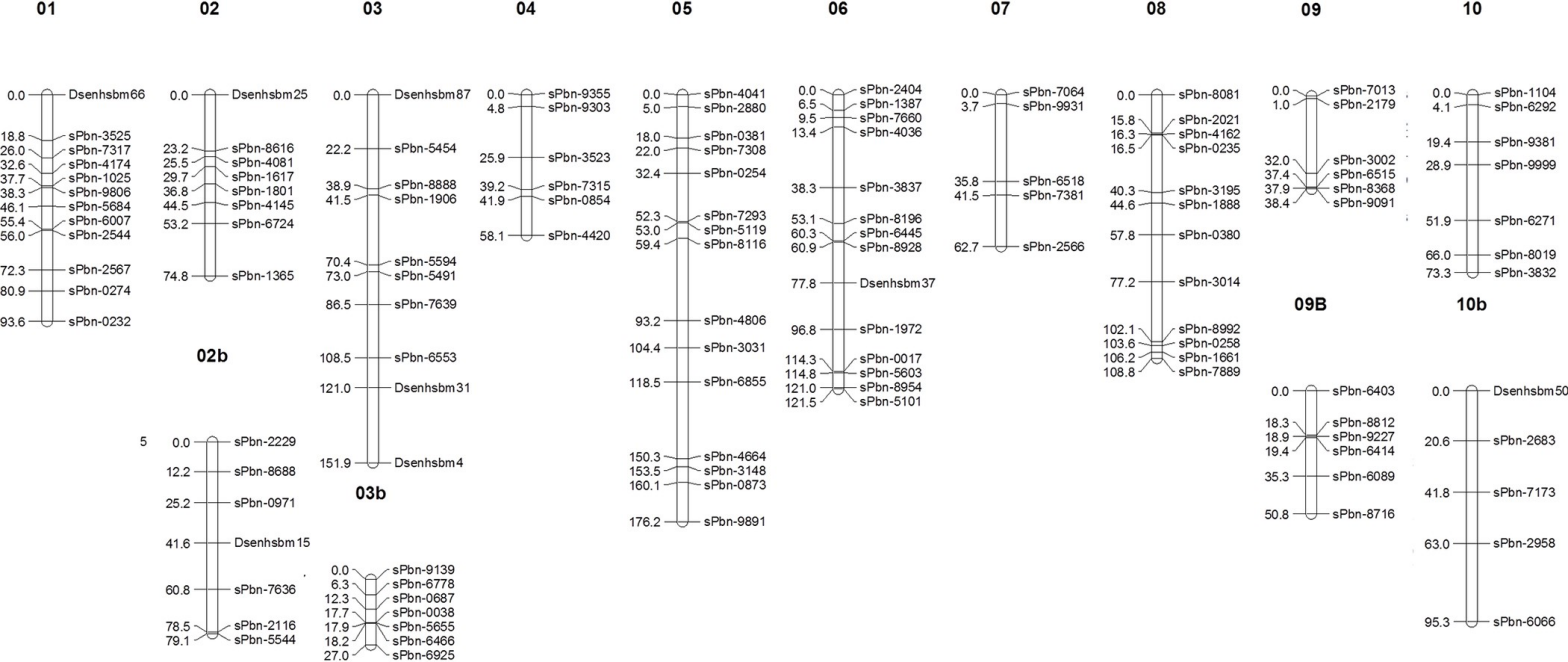
Genetic linkage map of the sorghum HYP x DTP RIL population. The map shows the positions of 112 DArT and 8 SSR markers distributed over 14 linkage groups corresponding to the 10 chromosomes of the *Sorghum bicolor* genome.

We constructed a genetic map with 14 linkage groups using a total of 120 markers, i.e. 112 DArTs and 8 SSRs markers. The map covered 1212 cM with an average marker distance of 10 cM (Fig 1 and S2 Table).

Means of the 18 measured traits of the parental lines and the RIL population are shown in Table 1. LA, TRL and RDW of parental lines showed antagonistic response to water availability, while HYP showed larger values in the control treatment, DTP showed larger values in the drought treatment. A similar situation was observed in case of HD since HYP was earlier in the control treatment while DTP was earlier under drought stress. HYP had higher SN, HSW and YLD than DTP in both treatments. Transgression beyond the two parents was observed for all traits. Heritability was moderate to high for all measured traits and ranged between 0.51 and 0.91. ANOVA showed significant GxE for LDW, SDW, AGDM, TRL and RDW in the first but not in the second experiment. HD, SN, HSW and YLD showed significant GxE in the third experiment.

**Table 1:** Parental lines and RILs performance for the analyzed traits.

Trait	Unit	Parental lines		RIL population					ANOVA		
		HYP	DTP	Min	Max	Mean	Std	h2	G	E	GxE
**LA_C1**	cm^2^	1425.43	959.06	656.70	1981.00	1350.00	254.20	0.55	0.361	<0.0001	0.1439
**LA_D1**	cm^2^	310.90	390.36	217.90	569.30	373.60	79.34	0.61			
**LA_C2**	cm^2^	604.41	538.39	221.30	1022.00	590.90	137.10	0.79	<0.0001	0.0007	0.3381
**LA_D2**	cm^2^	526.29	551.81	141.20	934.20	546.90	124.10	0.66			
**LDW_C1**	G	5.79	3.84	3.24	8.41	6.27	0.99	0.64	<0.0001	<0.0001	0.0038
**LDW_D1**	G	1.91	1.50	1.43	2.60	2.14	0.20	0.63			
**LDW_C2**	G	1.97	1.90	0.68	3.64	2.05	0.48	0.80	<0.0001	0.0023	0.2219
**LDW_D2**	G	2.03	2.08	0.68	3.30	1.90	0.45	0.70			
**SDW_C1**	G	3.46	2.46	2.47	5.72	4.03	0.72	0.66	<0.0001	<0.0001	0.0112
**SDW_D1**	G	1.22	1.03	0.83	2.11	1.52	0.25	0.73			
**SDW_C2**	G	1.92	1.76	0.34	3.52	1.93	0.58	0.67	<0.0001	0.0005	0.7943
**SDW_D2**	G	2.05	1.34	0.60	3.26	1.72	0.51	0.78			
**AGDM_C1**	G	8.42	6.56	6.01	13.35	10.29	1.62	0.64	<0.0001	<0.0001	0.0210
**AGDM_D1**	G	3.13	3.65	2.42	4.53	3.65	0.39	0.76			
**AGDM_C2**	G	3.89	3.67	1.02	5.91	3.98	0.89	0.70	<0.0001	<0.0001	0.4565
**AGDM_D2**	G	3.85	4.00	1.34	5.25	3.62	0.77	0.66			
**SLA_C1**	cm^2^ g^-1^	246.05	250.08	142.10	267.50	216.60	28.63	0.51	<0.0001	0.1535	0.3665
**SLA_D1**	cm^2^ g^-1^	162.77	260.53	100.80	273.50	175.50	36.80	0.53			
**SLA_C2**	cm^2^ g^-1^	307.39	283.17	207.90	343.20	290.80	21.91	0.52	<0.0226	0.5185	0.0873
**SLA_D2**	cm^2^ g^-1^	259.10	265.77	225.16	503.20	364.18	42.94	0.58			
**TRL_C1**	Cm	4127.61	3912.36	2806.00	10220.00	6049.00	1826.00	0.77	<0.0001	<0.0001	<0.0001
**TRL_D1**	Cm	4721.26	4768.22	2785.00	6271.00	4729.00	683.60	0.54			
**TRL_C2**	Cm	4493.89	4268.17	1561.00	7530.00	4629.00	919.10	0.67	<0.0001	<0.0001	0.1323
**TRL_D2**	Cm	4983.85	5457.45	2549.00	6321.00	4910.00	746.10	0.61			
**RDW_C1**	G	2.36	1.75	1.11	3.82	2.35	0.65	0.72	<0.0001	<0.0001	0.0442
**RDW_D1**	G	1.53	1.69	0.85	2.79	1.69	0.39	0.63			
**RDW_C2**	G	0.77	0.69	0.12	1.25	0.69	0.19	0.67	<0.0001	<0.0001	0.4574154
**RDW_D2**	G	0.94	1.23	0.34	1.23	0.81	0.19	0.64			
**HD_C3**	DAS	65	59	51.5	80	64.8	6.7	0.74	<0.0001	0.0100	0.0200
**HD_D3**	DAS	61.5	60	47	79.5	61.4	7.2	0.76			
**SN_C3**	-	903.31	425.09	100.00	1527.49	667.75	284.46	0.74	<0.0001	<0.0001	0.0831
**SN_D3**	-	536.64	491.42	98.77	759.33	388.98	154.26	0.71			
**HSW_C3**	G	2.07	1.52	0.24	3.59	1.46	0.81	0–88	<0.0001	<0.0001	0.0524
**HSW_D3**	G	1.97	1.37	0.19	3.51	1.72	0.74	0.91			
**YLD_C3**	g plant^-1^	17.72	6.96	0.63	23.225	12.084	6.4597	0.82	<0.0001	<0.0001	0.0125
**YLD_D3**	g plant^-1^	10.44	6.64	0.19	10.07	5.02	2.37	0.76			

The letters C and D after trait names refer to the control and drought stress conditions in the three experiments 1,2 and 3. HYP = high yielding parent, DTP = drought tolerant parent, LA = leaf area, LDW = leaf dry weight, SDW = stem dry weight, AGDW = above ground dry weight, SLA = specific leaf area, TRL = total root lengths, RDW = root dry weight, HD = heading date, HSW = hundred seed weight, SN = Seed number per plant, YLD = yield. Std is the standard deviation and h^2^ the heritability of the analyzed traits. For ANOVA results, statistical significance is assumed if P < 0.05 according to mixed linear model results with random genotype (G), fixed treatment (E) and genotype by treatment interaction effects (GxE).

**Fig 2:**
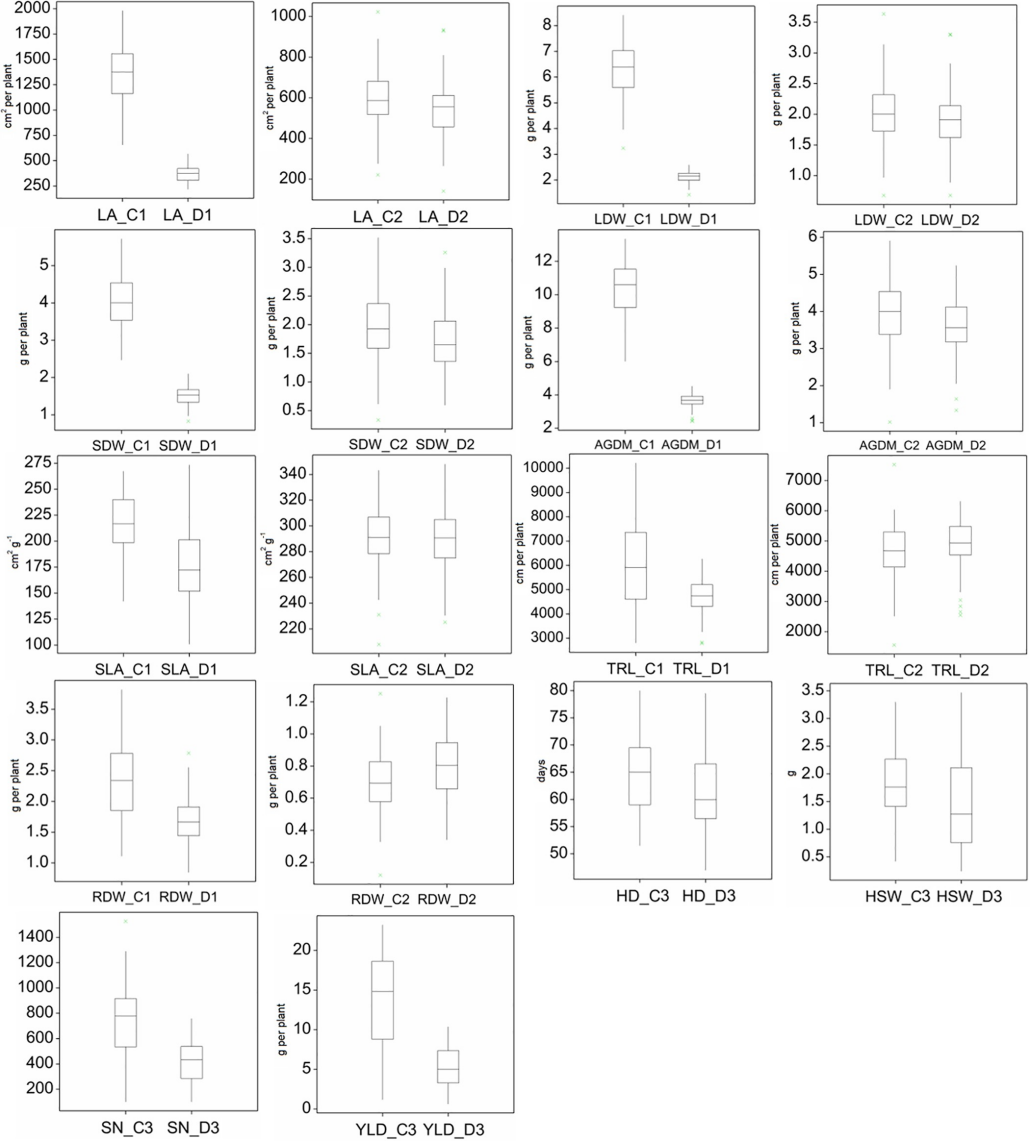
Boxplots of the measured traits, leaf area (LA), leaf dry weight (LDW), shoot dry weight (SDW), Above ground dry matter (AGDM), specific leaf area (SLA), total root lengths (TRL), root dry weight (RDW), heading date (HD), hundred seed weight (HSW), seed number (SN) and yield (YLD) of the sorghum RIL-population in the control (C) and drought stress (D) treatments of experiments 1, 2 and 3. Boxes represent the median and the upper and lower quartile, the maximum and minimum. Outliers were only removed if the value was < mean– 3*standard deviation or > mean + 3*standard deviation of the respective treatment/trait combination.

Fig 2 display the effects of drought stress on the performance of the RIL population. As can be seen from Fig 2, drought stress effects on vegetative plant growth, i.e. LDW, SDW and AGDM, were more severe during experiment 1.

Correlation analysis revealed positive significant correlations between most of the traits (Tables 2, 3 and 4). TRL was positively correlated with LA, LDW, SDW and AGDM, while SLA was positively correlated with LA in both treatments of the first and second experiment. SN and HD showed negative correlations in the control treatment, while they were positively correlated in the drought treatment. SN was negatively correlated with HSW and positively correlated with YLD in both treatments.

**Table 2:** Pearson’s correlation coefficients between traits measured in experiment 1.

	LA_C	LDW_C	SDW_C	AGDM_C	SLA_C	TRL_C	RDW_C	LA_D	LDW_D	SDW_D	AGDM_D	SLA_D	TRL_D
LA_C	1												
LDW_C	.722**	1											
SDW_C	.583**	.791**	1										
AGDM_C	.700**	.962**	.928**	1									
SLA_C	.545**	-.173	-.127	-.162	1								
TRL_C	.493**	.682**	.599**	.683**	-.101	1							
RDW_C	.591**	.739**	.666**	.747**	-.024	.692**	1						
LA_D	-.165	-.219*	-.130	-.191	.066	-.124	-.221*	1					
LDW_D	.134	.127	.081	.114	.026	-.088	-.056	.228*	1				
SDW_D	.107	.191	.333**	.265**	-.109	.104	.130	-.103	.545**	1			
AGDM_D	.136	.185	.250*	.224*	-.055	.020	.053	.051	.851**	.904**	1		
SLA_D	-.225*	-.268**	-.166	-.237*	.039	-.079	-.203*	.871**	-.255*	-.369**	-.361**	1	
TRL_D	.221*	.100	.011	.066	.225*	.063	.107	.149	.325**	.150	.259**	-.032	1
RDW_D	.321**	.308**	.085	.226*	.116	.245*	.353**	-.235*	.169	.100	.149	-.324**	.356**

Traits were analyzed in experiment 1 including a control (C) and a drought stress (D) treatment. LA = leaf area, LDW = leaf dry weight, SDW = stem dry weight, AGDM = above ground dry matter, SLA = specific leaf area, TRL = total root length, RDW = root dry weight, HD = heading date, HSW = hundred seed weight, SN = Seed number per plant, YLD = yield. Statistically significant correlations between traits are displayed at the 0.05

(*) and 0.01

(**) probability level.

**Table 3:** Pearson’s correlation coefficients between traits measured in experiment 2.

	LA_C	LDW_C	SDW_C	AGDM_C	SLA_C	TRL_C	RDW_C	LA_D	LDW_D	SDW_D	AGDM_D	SLA_D	TRL_D
LA_C	1												
LDW_C	.939**	1											
SDW_C	.360**	.418**	1										
AGDM_C	.738**	.808**	.873**	1									
SLA_C	.152	-.186	-.212*	-.237*	1								
TRL_C	.468**	.515**	.758**	.768**	-.188	1							
RDW_C	.528**	.597**	.777**	.824**	-.261**	.779**	1						
LA_D	.651**	.672**	.235*	.513**	-.048	.273**	.252*	1					
LDW_D	.614**	.662**	.115	.430**	-.100	.228*	.206*	.859**	1				
SDW_D	.055	.073	.575**	.412**	-.056	.372**	.297**	.262**	.284**	1			
AGDM_D	.397**	.437**	.447**	.524**	-.096	.380**	.317**	.677**	.775**	.827**	1		
SLA_D	.029	.017	.177	.124	-.003	.086	.078	.277**	-.177	.016	-.094	1	
TRL_D	.345**	.400**	.435**	.497**	-.153	.418**	.376**	.514**	.510**	.614**	.704**	.129	1
RDW_D	.254*	.317**	.374**	.413**	-.149	.254*	.276**	.485**	.561**	.714**	.801**	.012	.744**

Traits were analyzed in experiment 2 including a control (C) and a drought stress (D) treatment. LA = leaf area, LDW = leaf dry weight, SDW = stem dry weight, AGDM = above ground dry matter, SLA = specific leaf area, TRL = total root length, RDW = root dry weight, HD = heading date, HSW = hundred seed weight, SN = Seed number per plant, YLD = yield. Statistically significant correlations between traits are displayed at the 0.05

(*) and 0.01

(**) probability level.

**Table 4:** (continued): Pearson’s correlation coefficients between traits measured in experiment 3.

	HD_C	SN_C	HKG_C	YLD_C	HD_D	SN_D	HSW_D
HD_C	1						
SN_C	-.252**	1					
HKG_C	-.637**	-.025	1				
YLD_C	-.582**	.732**	.617**	1			
HD_D	.811**	-.247**	-.765**	-.678**	1		
SN_D	.340**	.249**	-.467**	-.188*	.343**	1	
HSW_D	-.887**	.100	.739**	.569**	-.797**	-.525**	1
YLD_D	-.609**	.425**	.377**	.552**	-.521**	.354**	.509**

Traits were analyzed in experiment 3 including a control (C) and a drought stress (D) treatment. LA = leaf area, LDW = leaf dry weight, AGDM = above ground dry matter, SDW = stem dry weight, SLA = specific leaf area, TRL = total root length, RDW = root dry weight, HD = heading date, HSW = hundred seed weight, SN = Seed number per plant, YLD = yield. Statistically significant correlations between traits are displayed at the 0.05

(*) and 0.01

(**) probability level.

### QTL mapping using the MTME approach

**Fig 3:**
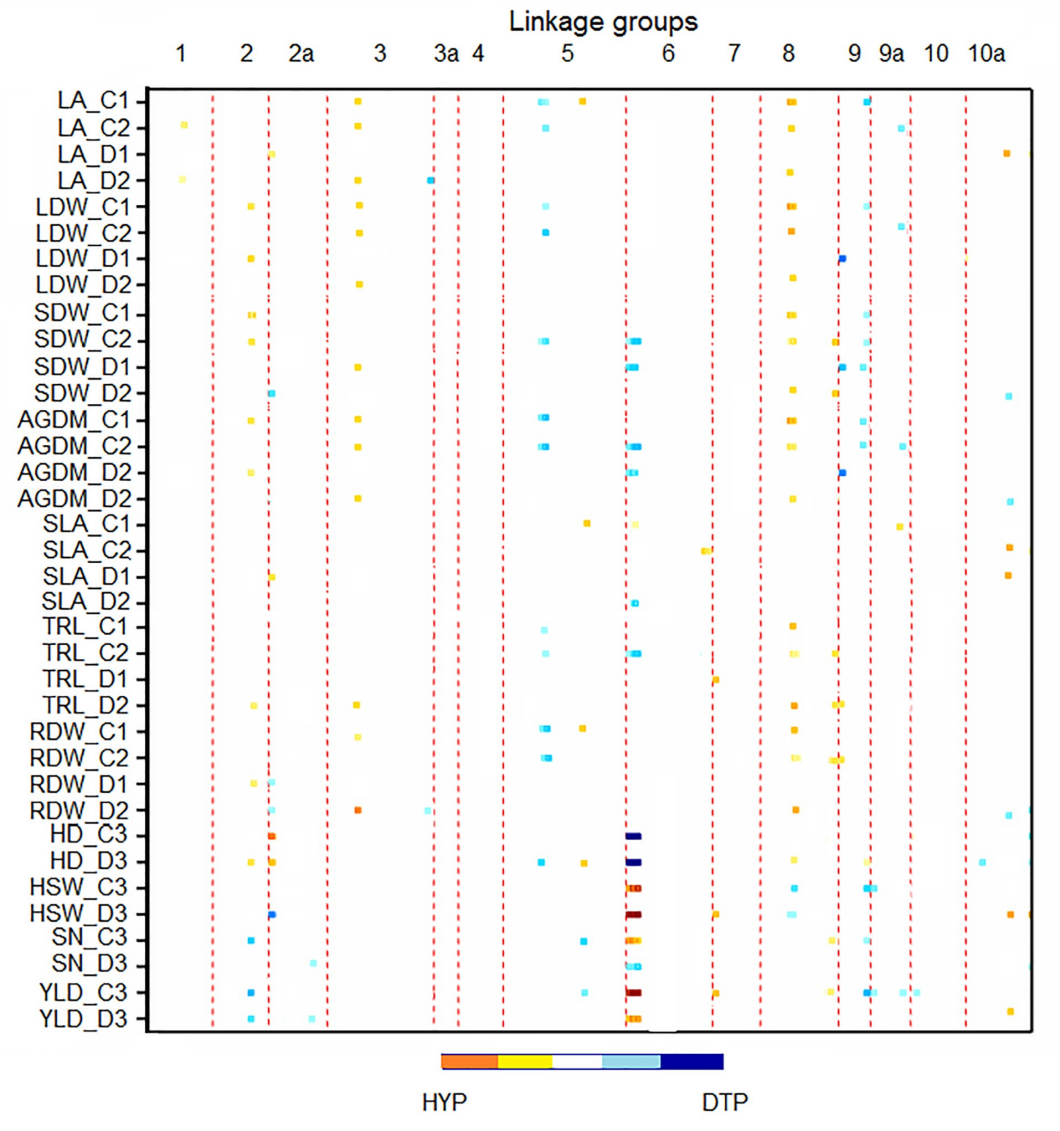
Heat map showing the QTL positions. The map represents the 14 linkage groups in columns and shows significant QTL across all trait-environment combinations using the multi-trait-multi-environment (MTME) approach. Light to dark blue indicates a significant positive effect from the DTP allele and yellow to red indicates a significant positive effect from the HYP allele (the darker the color, the higher the significance). C and D refer to control and drought treatments, respectively. The numbers 1, 2, and 3 refer to the three experiments. LA refers to leaf area, LDW to leaf dry weight, SDW = stem dry weight, AGDM to above ground dry matter, SLA is specific leaf area, TRL refers to total root length, RDW to root dry weight, HD to heading date, HSW is hundred seed weight, SN refers to seed number, and YLD to yield.

In total, 133 significant QTL (*p* < 0.05) were detected in ten hotspot regions and mapped mainly to LGs 2, 2a, 3, 5, 6, 8, 9, 9a, and 10a (Fig 3, Tables 5 and 6).

**Table 5:** QTL mapped on linkage groups 1 to 6 in the HYP x DTP recombinant inbred line population.

LG	Pos	Marker	-LOG10	Trait	Effect	R^2^	LG	Pos	Marker	-LOG10	Trait	Effect	R^2^
1	46.1	sPbn-5684	6.5	LA_C2	-0.203	4.1	3	151.9	Dsenhsbm4	5.211	LA_D2	0.299	9
				LA_D2	-0.218	4.7					RDW_D2	0.295	8.7
2	53.2	sPbn-6724	9.904	LDW_C1	-0.302	9.1	5	59.4	sPbn-8116	7.265	LA_C1	0.334	11.1
				LDW_D1	-0.25	6.2					LA_C2	0.329	10.8
				SDW_C1	-0.261	6.8					LDW_C1	0.286	8.2
				SDW_C2	-0.183	3.4					LDW_C2	0.32	10.2
				AGDM_C1	-0.296	8.7					SDW_C2	0.205	4.2
				AGDM_D1	-0.219	4.8					AGDM_C1	0.224	5
				TRL_D2	-0.245	6					AGDM_C2	0.283	8
				RDW_D1	-0.238	5.7					TRL_C1	0.213	4.6
				HD_D3	-0.163	2.7					TRL_C2	0.239	5.7
				SN_C3	0.266	7.1					RDW_C1	0.279	7.8
				YLD_C3	0.244	6					RDW_C2	0.325	10.6
				YLD_D3	0.18	3.3					HD_D3	0.143	2
2a	0	sPbn-2229	15.392	LA_D1	-0.318	10.1	5	118.5	sPbn-6855	7.787	LA_C1	-0.274	7.5
				SDW_D2	0.309	9.6					SLA_C1	-0.233	5.4
				SLA_D1	-0.319	10.2					RDW_C1	-0.23	5.3
				RDW_D1	0.229	5.2					HD_D	-0.145	2.1
				RDW_D2	0.211	4.4					SN_C	0.206	4.2
				HD_C3	-0.231	5.3					YLD_C	0.206	4.2
				HD_D3	-0.179	3.2	6	9.5	sPbn-7660	32.171	SDW_C2	0.311	9.7
				HSW_D3	0.235	5.5					SDW_D1	0.247	6.1
2a	60.8	sPbn-7636	14.391	SN_D3	0.193	3.7					AGDM_C2	0.251	6.3
				YLD_D3	0.193	3.7					AGDM_D1	0.223	5
3	41.5	sPbn-1906	4.716	LA_C1	-0.298	8.9					SLA_C1	-0.193	3.7
				LA_C2	-0.327	10.7					SLA_D2	0.31	9.6
				LA_D2	-0.342	11.7					TRL_C2	0.274	7.5
				LDW_C1	-0.239	5.7					HD_C3	0.446	19.9
				LDW_C2	-0.347	12					HD_D3	0.448	20.1
				LDW_D2	-0.379	14.4					HSW_C3	-0.342	11.7
				SDW_D1	-0.286	8.2					HSW_D3	-0.393	15.5
				AGDM_C1	-0.216	4.7					SN_C3	-0.215	4.6
				AGDM_C2	-0.248	6.1					SN_D3	0.218	4.7
				AGDM_D2	-0.328	10.7					YLD_C3	-0.363	13.2
				TRL_D2	-0.262	6.9					YLD_D3	-0.257	6.6
				RDW_C1	-0.26	6.8	6	114.3	sPbn-0017	8.921	SLA_C2	-0.231	5.3
				RDW_D2	-0.387	15							

QTL were mapped using the multi-trait multi-environment analysis. LG refers to linkage group and pos refers to marker position in cM. *R*^2^ is the percentage of total phenotypic variance explained by each QTL. Effects with positive values represent a positive contribution of the DTP allele to the trait, while negative values represent a positive contribution of the HYP allele. C and D refer to the control and drought treatments, respectively. Numbers 1, 2, and 3 refer to the three experiments. LA = leaf area, LDW = leaf dry weight, SDW = stem dry weight, AGDM = above ground dry matter, SLA = specific leaf area, TRL = total root length, RDW = root dry weight, HD = heading date, HSW = hundred seed weights, SN = seed number and YLD = yield.

**Table 6:** QTL mapped on linkage groups 7 to 10 in the HYP x DTP recombinant inbred line population.

LG	Pos	Marker	-LOG10	Trait	Effect	R^2^	LG	Pos	Marker	-LOG10	Trait	Effect	R^2^
7	0	sPbn-7064	13.091	TRL_D1	-0.288	8.3	9	1	sPbn-2179	4.039	LDW_D1	0.333	11.1
				HSW_D3	-0.206	4.2					SDW_D1	0.242	5.9
				YLD_C3	-0.169	2.9					AGDM_D1	0.315	9.9
8	57.8	sPbn-0380	7.123	LA_C1	-0.28	7.8					TRL_D2	-0.212	4.5
				LA_C2	-0.292	8.5					RDW_C2	-0.205	4.2
				LA_D2	-0.316	10	9	38.4	sPbn-9091	7.526	LA_C1	0.324	10.5
				LDW_C1	-0.429	18.4					LDW_C1	0.289	8.4
				LDW_C2	-0.29	8.4					SDW_C1	0.256	6.6
				LDW_D2	-0.344	11.8					SDW_C2	0.221	4.9
				SDW_C1	-0.329	10.8					SDW_D1	0.226	5.1
				SDW_C2	-0.296	8.8					AGDM_C1	0.288	8.3
				SDW_D2	-0.275	7.6					AGDM_C2	0.231	5.3
				AGDM_C1	-0.402	16.2					TRL_D2	0.2	4
				AGDM_C2	-0.341	11.6					RDW_C2	0.255	6.5
				AGDM_D2	-0.374	14					HSW_C3	0.216	4.7
				TRL_C1	-0.417	17.4					YLD_C3	0.207	4.3
				TRL_C2	-0.297	8.8	9a	35.3	sPbn-6089	6.469	LA_C2	0.2	4
				TRL_D2	-0.425	18					LDW_C2	0.21	4.4
				RDW_C1	-0.234	5.5					AGDM_C2	0.199	4
				RDW_C2	-0.246	6					SLA_C1	-0.236	5.6
				RDW_D2	-0.291	8.5					YLD_C3	0.188	3.5
				HD_D3	-0.153	2.3	10a	20.6	sPbn-2683	4.588	HD_D3	0.165	2.7
				HSW_C3	0.296	8.8	10a	95.3	sPbn-6066	5.829	LA_D1	-0.375	14
8	108.8	sPbn-7889	4.303	SDW_C2	-0.224	5					SDW_D2	0.323	10.5
				SDW_D2	-0.218	4.7					AGDM_D2	0.217	4.7
				TRL_C2	-0.278	7.7					SLA_C2	-0.28	7.9
				TRL_D2	-0.174	3					SLA_D1	-0.328	10.8
				RDW_C2	-0.177	3.1					RDW_D2	0.26	6.8
				SN_C3	-0.185	3.4					HSW_D3	-0.222	4.9
				YLD_C3	-0.153	2.3					YLD_D3	-0.159	2.5

QTL were mapped using the multi-trait multi-environment analysis. LG refers to linkage group and pos refers to marker position in cM. *R*^2^ is the percentage of total phenotypic variance explained by each QTL. Effects with positive values represent a positive contribution of the DTP allele to the trait, while negative values represent a positive contribution of the HYP allele. C and D refer to the control and drought treatments, respectively. Numbers 1, 2, and 3 refer to the three experiments. LA = leaf area, LDW = leaf dry weight, SDW = stem dry weight, AGDM = above ground dry matter, SLA = specific leaf area, TRL = total root length, RDW = root dry weight, HD = heading date, HSW = hundred seed weights, SN = seed number and YLD = yield.

QTL of a hotspot on LG-3 had positive effects from the HYP allele for LA_C1, LA_C2, LA_D2, LDW_C1, LDW_C2, LDW_D2, SDW_D1, AGDM_C1, AGDM_C2, AGDM_D2, RL_D2, RDW_C1, and RDW_D2. The hotspot showed conditional neutrality for some traits since no QTL were mapped under drought stress in the first experiment for LA_D1, LDW_D1 and AGDM_D1. The main effect QTL cluster on top of LG-5 had a positive effect from DTP for LA_C1, LA_C2, LDW_C1, LDW_C2, SDW_C2, AGDM_C1, AGDM_C2, TRL_C1, TRL_C2, RDW_C1, RDW_C2, and HD_D3. A conditional neutrality for several traits was observed here as well since no QTL were found for LA, LDW, AGDM, TRL, and RDW in the drought treatment. In another cluster on top of LG-6, the DTP allele had positive effects on SDW_C2, SDW_D1, AGDM_C2, AGDM_D1, SLA_D2, TRL_C2, HD_C3, HD_D3, and SN_D3, while the HYP allele had positive effects on SLA_C1, HSW_C3, HSW_D3, SN_C3, YLD_C3, and YLD_D3. Within this cluster, an antagonistic effect was observed for SN since the positive effect was from the DTP allele in the drought and from the HYP allele in the control treatment. Considering the opposite allelic effects on different traits revealed antagonistic pleiotropic effects between HD and both HSW and YLD since the DTP allele had a positive effect on HD and the HYP allele increased HSW and YLD in both environments.

## Discussion

The population used here was genotyped with 120 DArT and SSR markers covering 14 LG and a total length of 1212 cM, which is comparable with the length of the sorghum consensus map, which had a size of 1355.4 cM [15]. DArT markers were used because they are affordable and represent a powerful high-throughput marker system suitable for QTL mapping. However, we are aware that the use of additional SNPs would be necessary to provide equal genome coverage and to allow direct comparisons with recent or future studies, in which SNPs are used [16–18].

Observing the performance of the two parents under drought stress revealed that both parents avoided drought by increasing their TRL and reducing their LA, for more water uptake and reduced transpiration. However, both parents did not escape drought by earliness. LA of the whole RIL population showed responses similar to the parental lines in both treatments of the first two experiments. In contrast to the two parents, the RIL population on average exhibited early heading in response to the drought treatment. Drought stress effects on vegetative plant growth, i.e. LDW, SDW and AGDM, were more severe during experiment 1, which is probably resulting from higher temperatures and radiation, since the experiment was carried out during spring/summer in Germany, while the third experiment was conducted in autumn. However, radiation and temperature were not explicitly measured during the experiments, so that we are not able to draw clear conclusions from these results.

Significant correlations between the measured traits allow to better understand response patterns to water availability. For example, plants heading early under drought stress reduced SN and increased HSW to maintain high YLD, a commonly observed mechanism and fitness tradeoff if stress occurs before or at anthesis. In contrast, plants heading early in the control treatment showed increased SN, HSW, and YLD, which are all desirable traits for selecting breeding lines. Such adaptive responses can be genetically dissected and explained by the antagonestic pleiotroic effects of the QTL clusters mapped to LG-2, LG-2a, and LG-6, which were identified since GxE was incorporated into the QTL model. For example, the DTP allele on LG-6 had positive effects on HD in both treatments and on SN in the drought treatment, whereas the HYP allele showed positive effects on HSW and YLD in both treatments and on SN in the control treatment.

Our results revealed significant G×E effects for all traits measured in the two treatments (control and drought) in the first and the third experiments but not in the second one. However, significant environment and genotype effects were observed for all measured traits in the second experiment. This was reflected by significant Q×E for all traits and enabled us to distinguish the QTL effects. For example, three QTL mapped for LA on top of LG-3, LG-5 and LG-8, showed conditional neutrality, they were associated with water availability in the first and second experiment.

We expected that the 10 QTL hotspots of the present study would overlap with previously mapped QTL for similar traits. To facilitate a comparison to previous studies, marker positions were compared based on the sorghum consensus map [15, 19]. An earlier study that used DArT markers [20] mapped a QTL for grain yield that was associated with sPb-3361 at 140.7 cM on chromosome 2. The QTL cluster on top of LG-2a was associated with the marker sPb-2229, which was mapped at 142.9 cM in the consensus map. The QTL cluster on LG-2a included QTL for several traits including HSW in the drought treatment. Another study detected a QTL for stay green and panicle length on Chromosome 2 [13]. Another QTL for chlorophyll fluorescence was mapped at DArT markers sPbn-2229 on LG-2a [21]. The QTL cluster on LG8 was associated with the marker sPb-7889 which mapped at 74 cM in the consensus map. A stay green QTL was associated with sPb-1661 which was mapped at 73.9 cM in the consensus map [20]. The QTL cluster on LG-9 overlapped with an earlier detected QTL for maturity [22] and another QTL with pleiotropic effects on flowering time and HSW [23]. We mapped QTL for several traits on LG9 at 52.5 and 140 cM according to the sorghum consensus map. These results indicate possible pleiotropic effects of the QTL on morphological traits and yield components, which were already proven in recent studies [24, 25]. The main QTL cluster on LG-6 was associated with the majority of traits measured in both water regimes. Earlier studies on sorghum reported a number of significant QTL on chromosome 6 that were associated with several traits measured under drought [26] as well as other environmental constraints such as thermal [27] and cold [18] stress and sorghum ergot [28]. Altogether, these results indicate the major role of chromosome 6 on sorghum growth and development under various environmental conditions making it an interesting target for future breeding programs.

Since the pot height used in the present study was much smaller than maximum rooting depth of sorghum plants, it was expected that investing additional energy in developing longer roots did not improve water uptake or increase YLD. Therefore, we assume that the negative correlation observed between TRL and both HSW and YLD in the drought treatment is an artifact of pot size.

## Conclusion

Understanding crop response to drought and the underlying QTL is essential to increase crop productivity under drought conditions which is the ultimate goal for breeding programs.

In that respect, mapping the QTL cluster on LG 6 with the observed antagonistic pleiotropic effects is very important to genetically dissect the significant antagonistic response by reducing SN and increasing HSW and YLD under drought conditions as an adaptive mechanism to cope with drought stress. In total, we detected 14 QTL clusters mapped on 11 LGs for the measured traits as a first step towards identifying genes governing those traits.

## Supporting information

S1 Table Phenotypic data used for QTL mapping. Trait appreviations are as following; Leaf area (LA), total root lengths (TRL), leaves dry weight (LDW), stems dry weight (SDW), roots dry weight (RDW), above ground dry matter (AGDM) was calculated as the sum of LDW and SDW, Specific leaf area (SLA) was calculated as ratio between LA and LDW, heading date (HD), seed number (SN), hundred seed weight (HSW) and yield (YLD).
(CSV)

S2 Table Marker positions on linkage groups. Positions in cM of the 120 DArT and SSR markers grouped in 14 linkage groups.
(TXT)
