# Correction: Unraveling the genetic complexity underlying sorghum response to water availability

**DOI:** 10.1371/journal.pone.0222859

**Published:** 2019-09-17

**Authors:** Nguyen Phuong, Gloria Afolayan, Hartmut Stützel, Ralf Uptmoor, Mohamed El-Soda

The following information is missing from the Funding statement: This study was supported by Deutsche Forschungsgemeinschaft and Universität Rostock within the funding programme Open Access Publishing (to RU).
